# Exercise Prevention of Cardiovascular Disease in Breast Cancer Survivors

**DOI:** 10.1155/2015/917606

**Published:** 2015-08-03

**Authors:** Amy A. Kirkham, Margot K. Davis

**Affiliations:** ^1^Rehabilitation Sciences, University of British Columbia, 212–2177 Wesbrook Mall, Vancouver, BC, Canada V6T 1Z3; ^2^Division of Cardiology, University of British Columbia, Diamond Health Care Centre, 9th Floor, 2775 Laurel Street, Vancouver, BC, Canada V5Z 1M9

## Abstract

Thanks to increasingly effective treatment, breast cancer mortality rates have significantly declined over the past few decades. Following the increase in life expectancy of women diagnosed with breast cancer, it has been recognized that these women are at an elevated risk for cardiovascular disease due in part to the cardiotoxic side effects of treatment. This paper reviews evidence for the role of exercise in prevention of cardiovascular toxicity associated with chemotherapy used in breast cancer, and in modifying cardiovascular risk factors in breast cancer survivors. There is growing evidence indicating that the primary mechanism for this protective effect appears to be improved antioxidant capacity in the heart and vasculature and subsequent reduction of treatment-related oxidative stress in these structures. Further clinical research is needed to determine whether exercise is a feasible and effective nonpharmacological treatment to reduce cardiovascular morbidity and mortality in breast cancer survivors, to identify the cancer therapies for which it is effective, and to determine the optimal exercise dose. Safe and noninvasive measures that are sensitive to changes in cardiovascular function are required to answer these questions in patient populations. Cardiac strain, endothelial function, and cardiac biomarkers are suggested outcome measures for clinical research in this field.

## 1. Introduction

Breast cancer is the most common malignancy among women worldwide [1], and an estimated 1% of the population are survivors of breast cancer [2]. Advances in breast cancer therapy have contributed to dramatic improvements in survival, but many of these therapies, particularly anthracycline chemotherapy, left-sided radiotherapy, and trastuzumab targeted therapy, are associated with cardiovascular toxicities [3]. Breast cancer survivors are at increased risk of cardiovascular disease-related death compared to women without breast cancer [4], likely due in part to these toxicities. An increased prevalence of traditional cardiovascular risk factors in this population at diagnosis, and lifestyle perturbations associated with cancer treatment also contribute to this increased risk [3]. Chemotherapy for breast cancer will induce menopause in one- to two-thirds of women [5], further increasing cardiovascular risk [1, 6]. As breast cancer survival rates rise, cardiovascular disease becomes an increasingly important competing risk [7]. Combined, these factors contribute to the recent finding that cardiovascular disease has surpassed breast cancer as the leading cause of death in older women diagnosed with breast cancer [8].

Current strategies to mitigate cardiotoxicity associated with anthracycline treatment include dose reduction, modified administration methods, liposomal formulations, and administration of cardioprotective medications [9]. However, dose modification may be associated with reduced oncological benefit [10], and pharmacological interventions may be associated with additional side effects.

Aerobic exercise training and other forms of physical activity are effective in primary and secondary prevention of cardiovascular disease and cardiovascular disease-related death [11]. For breast cancer survivors, exercise training is safe and effective in improving cardiorespiratory fitness, strength, body composition, fatigue, anxiety, depression, and quality of life, and is recommended during and after treatment [12]. However, the effect of aerobic exercise on cardiovascular function and outcomes during or after breast cancer treatment is not well established in humans.

The purpose of this paper is to (1) review the potential mechanisms mediating exercise prevention of cardiovascular toxicity; (2) review the available evidence for the role of exercise in prevention of cardiovascular disease in breast cancer survivors, including predominantly preclinical studies of the heart and clinical studies of cardiovascular risk factors; and (3) suggest outcome measures for translation of the preclinical findings to clinical studies.

## 2. Potential Mechanisms Mediating Exercise Prevention of Cardiovascular Toxicity

The vast majority of studies investigating exercise prevention of direct cardiovascular toxicity are in rodent models utilizing the anthracycline agent doxorubicin and compare an exercise-trained treated group to a sedentary treated group. The discussion of mechanisms and preclinical evidence refers to studies with this design unless otherwise noted. The mechanism underlying the cardioprotective effects of aerobic exercise before or during treatment with doxorubicin has not been fully elucidated but is likely to be multifactorial with summative effects and feedback from diverse processes. Potential mechanisms by which exercise may act in opposition to the negative effects of doxorubicin to protect the heart and vasculature are listed in Table 1. There is available evidence for exercise protection mechanisms related to reduced oxidative stress, interruption of topoisomerase-mediated pathways, cardiomyocyte contractile protein isoform shifts, and upregulation of heat shock proteins (HSP), endothelial nitric oxide (NO), and endothelial progenitor cells.

The most widely supported mechanism by which exercise may prevent doxorubicin cardiotoxicity is through its antioxidant effects. The production of reactive oxygen species (ROS) is one of the possible mechanisms for doxorubicin cardiotoxicity [13, 14]. Although cells are equipped with an endogenous antioxidant system to protect against ROS, cardiomyocytes have only one fourth of the antioxidative capacity of the liver and other tissues [15], making them particularly vulnerable to oxidative stress. Exercise-induced enhancement of cardiomyocyte antioxidant capacity may prevent ROS-induced damage associated with doxorubicin treatment [16]. Compared with untrained animals, exercise-trained rodents have increased levels of antioxidant activity and reduced levels of oxidative stress markers following doxorubicin exposure [17–22]. However this mechanism may not play a role in cardioprotection when exercise is of low intensity and duration [23]. Reduced levels of protein turnover via the ubiquitin-proteasome pathway, an important mechanism for degradation of cellular proteins with oxidative damage, have been demonstrated in exercise-trained rodents compared to sedentary rodents [24]. This finding provides further support for exercise protection via reduced oxidative stress.

Anthracycline-induced ROS cause lipid peroxidation [25] and downregulate expression of the sarcoplasmic reticulum calcium pump, SERCA2a [14]. Decreased calcium uptake by SERCA2a then leads to an increase in cytosolic calcium [14]. These two changes result in opening of the mitochondrial permeability transition pore, allowing release of calcium from the mitochondrial matrix, downregulation of mitochondrial respiration, and leaking of proapoptotic mitochondrial proteins into the cytosol [26, 27]. A single submaximal exercise session 24 hours before doxorubicin treatment prevented opening of the mitochondria permeability transition pore, mitigating the downstream effects [26]. This hypothesis is supported indirectly by several other studies demonstrating attenuation of doxorubicin-associated increases in the proapoptotic proteins caspase-9 and 3 in exercise trained rodents [18, 23, 24, 26]. These findings may be related to modulation of defense systems including stress chaperones like HSPs, or antioxidants, but may not be related to exercise-induced upregulation of SERCA2a [28, 29].

There is emerging evidence implicating topoisomerase 2*β*, an enzyme regulating DNA unwinding, in doxorubicin-induced cardiomyocyte mitochondrial dysfunction [30], secondary to downregulation of peroxisome proliferator-activated receptor-*γ* coactivator (PGC)-1*α*, a transcriptional coactivator of mitochondrial biogenesis [31]. Exercise training upregulates expression of PGC-1*α* in skeletal muscle, although a similar response in cardiomyocytes has not been observed [32, 33]. Two recent preclinical studies investigating the role of PGC-1*α* in exercise cardioprotection did not demonstrate an interaction between exercise and doxorubicin [22, 34]. However, the capacity of exercise to impact topoisomerase 2*β* and PGC-1*α* in cardiomyocytes requires further investigation before this mechanism can be dismissed.

In the rodent heart, doxorubicin causes disruption of cardiac bioenergetics and an associated shift from the *α* isoform of the contractile protein, myosin heavy chain (MHC), to the *β* isoform which has reduced contractile power [35]. Exercise training before [28, 35] and during doxorubicin treatment [36, 37] conserves the *α* isoform in rats. However, healthy human hearts express 7% of the *α* isoform on average, while this is the predominant isoform expressed in the rat heart [38]. Therefore the extent and subsequent impact of a doxorubicin-induced shift in MHC isoform distribution may be smaller for human myocardium. Clinical research is required to clarify the role of prevention of MHC isoform shifts in exercise cardioprotection.

HSPs control protein folding and unfolding, and are upregulated in cardiomyocytes during times of oxidative stress [24]. An exercise-induced increase in HSP expression is hypothesized to play a role in cardioprotection against doxorubicin by preserving the integrity and activity of mitochondrial respiratory complexes and thereby attenuating mitochondrial dysfunction [39]. Although there is some evidence supporting HSP-mediated cardioprotection [17, 19, 23], there are also conflicting results [19, 24, 40].

Breast cancer therapies, including chemotherapy, targeted therapies, and radiotherapy, may be associated with endothelial dysfunction, a disease process involving impaired regulation of vascular tone and loss of atheroprotection [41]. Flow-mediated dilatation is triggered by shear stress from increased blood flow through a vessel, resulting in NO-mediated vasodilation [42]. Doxorubicin impairs both endothelium-dependent (i.e., flow-mediated) and endothelium-independent vasodilation [41, 43, 44]. Breast radiation impairs endothelium-dependent vasodilation in exposed axillary arteries, causes ultrastructural damage to myocardial capillaries, and can induce atherosclerosis in coronary arteries [45–48]. Trastuzumab may cause endothelial dysfunction through reductions in NO [49].

Exercise training improves endothelial dysfunction, predominantly through increased NO production as a result of chronic periods of pulsatile blood flow [50]. In the presence of the superoxide ROS, NO reacts to form a reactive molecule that can damage DNA, and this reaction also decreases the bioavailability of NO [51]. The upregulation of antioxidative enzymes associated with exercise training may therefore promote NO bioavailability by scavenging ROS [51]. Hayward et al. provided evidence that exercise preconditioning prior to 5-fluorouracil chemotherapy exposure increased NO production in rodents [52].

Endothelial progenitor cells (EPCs) contribute to maintaining the integrity of the endothelial cell layer, and lower levels of circulating EPCs are associated with an increased risk of cardiovascular events and death [53]. Exercise stimulates EPC mobilization from the bone marrow [54]. In human breast cancer survivors receiving doxorubicin-containing chemotherapy, exercise has been associated with an increase in circulating EPCs relative to usual care controls [55].

There are other proposed mechanisms for cardiotoxicity where exercise training could counteract the doxorubicin-induced molecular response that have not yet been investigated as mechanisms for exercise cardioprotection. For example, pharmacological *α*1-adrenoceptor activation of the cardiac transcription factor GATA-4 has demonstrated cardioprotective capacity against doxorubicin [56]. Therefore, exercise training, which appears to enhance both *α*1-adrenoceptor responsiveness [57], and GATA-4 mRNA level in the heart [58] may also exert a cardioprotective effect via a GATA-4 pathway. Another example includes doxorubicin and trastuzumab downregulation of neuregulin-1/ErbB4 receptor tyrosine kinase signaling in cardiomyocytes. Neuregulin-1/ErbB4 signaling plays a critical role in cardiac development and cardiomyocyte survival and organization [59]. Intriguingly, exercise training upregulates expression of neuregulin-1 in rodent cardiomyocytes [60], indicating a potential mechanism for exercise prevention of doxorubicin- and trastuzumab-related cardiotoxicity. Readers are referred to a more comprehensive review of potential mechanisms for exercise prevention of targeted cancer therapy-related cardiotoxicity [61].

In summary, although evidence exists for several different mechanisms through which exercise protects the heart and vasculature from doxorubicin-related toxicity, the unifying feature appears to be increased antioxidant capacity and reduction of oxidative stress. Several potential mechanisms, including exercise-induced upregulation of topoisomerase 2*β*/PGC-1*α*, GATA-4, and neuregulin-1/ErbB4 warrant further investigation to determine their role in cardioprotection.

## 3. Evidence for Exercise Prevention of Cardiovascular Disease

### 3.1. Cardiotoxicity Prevention

#### 3.1.1. Acute Exercise

In animal models, doxorubicin-related cardiotoxicity can be attenuated by a single exercise session in close proximity to time of exposure. In the seminal study in this area, a 30-minute exercise session completed half an hour after doxorubicin exposure reduced mortality [62]. These findings were extended to demonstrate that an exhaustive exercise session half an hour after doxorubicin exposure attenuated markers of cardiomyocyte mitochondrial dysfunction [63]. Sixty minutes of submaximal exercise performed 24 hours prior to doxorubicin prevented or attenuated left ventricular (LV) systolic and diastolic dysfunction, cardiomyocyte mitochondrial apoptosis and dysfunction, and lipid peroxidation at 5 days post-treatment in rodents [26, 64].

The potential of a single exercise session to provide cardioprotection is particularly appealing, as regular, supervised exercise training during chemotherapy may not be feasible for all patients due to distance from home to exercise centers, difficulty with treatment symptoms, scheduling conflict with work, or family obligations. Ongoing research by our group is investigating the cardioprotective benefit of an acute exercise session 24 hours prior to doxorubicin administration in women with breast cancer.

#### 3.1.2. Exercise Training before Treatment

In animals receiving high-dose bolus doxorubicin, exercise preconditioning prevents or attenuates acute (~24 hour post) increases in cardiac troponin I [17, 18], markers of oxidative stress [17–21, 24, 65], cardiomyocyte mitochondrial dysfunction [18, 19, 24], morphological and histological damage [16], markers of apoptosis [18, 24, 66], and decreases in HSP expression [17, 19] and LV systolic function [40, 65]. Similar findings have been reported in studies that extended the follow-up time to 5–10 days after doxorubicin exposure [35, 40, 67, 68]. Findings exclusive to studies with longer follow-up include attenuation of deficits in coronary flow [40], transmitral, and transaortic flow [35, 67], as well as transformation to the *β*-MHC isoform [35]. Even at four weeks after doxorubicin exposure, the beneficial effects of exercise preconditioning on *β*-MHC transformation, LV wall thickness, mass and systolic function, and transmitral/transaortic flow were still apparent [28].

The feasibility of exercise preconditioning in humans has been questioned, as the interval between breast cancer diagnosis and treatment is shorter than the length of most training programs that have been studied (8 to 14 weeks). However, cardioprotective effects have been reported after as little as 5 days to 3 weeks of training in rodents [21, 24, 66]. It should be noted that administered doxorubicin doses in these studies were higher than comparable human doses. It is unclear whether similar benefits would be seen in patients receiving standard treatment doses.

#### 3.1.3. Exercise Training during Treatment

Exercise training concurrent to chronic doxorubicin treatment in rodents has been associated with attenuation of LV systolic and diastolic dysfunction [23, 29, 37, 69, 70], cardiomyocyte apoptosis [23], transformation to *β*-MHC [36, 37], reductions in LV wall thickness [69] and heart mass [22], and deficits in coronary [23], transmitral, and transaortic flow [29, 37, 69].

Exercise training in humans during chemotherapy treatment for breast cancer is feasible and prevents the decrease in cardiorespiratory fitness seen in usual care controls [70–72]. Preliminary clinical studies of the effects of exercise training on cardiac function in humans undergoing breast cancer treatment have had disappointing results, however. A small randomized control trial of exercise training compared to usual care during doxorubicin-containing chemotherapy for breast cancer found no change in LV ejection fraction (LVEF) in either group [70]. A single-arm study investigated the effects of four months of exercise training in 17 breast cancer survivors receiving adjuvant trastuzumab therapy. Despite exercise training, trastuzumab was associated with LV dilatation and reduced LVEF [73]. However the exercise training dose may have been insufficient, as participants did not attend 41% of exercise sessions. More sensitive measures of cardiac function and a higher exercise dose are likely required in order to demonstrate a cardioprotective benefit in clinical studies.

#### 3.1.4. Exercise Training after Treatment

Although Héon et al. have reported reduced markers of cardiomyocyte apoptosis and oxidative stress in rodents undergoing exercise training two weeks after the completion of doxorubicin administration [74], to our knowledge the effects of post-treatment exercise on cardiac function have not been studied.

#### 3.1.5. Summary of Cardiotoxicity Prevention Evidence

In summary, acute and chronic exercise before, during or after doxorubicin treatment in rodents consistently results in prevention or attenuation of doxorubicin-induced deleterious effects to cardiomyocyte morphology and biochemistry, as well as cardiac function. Preclinical experimental research is needed to determine whether exercise can provide cardioprotection from cancer therapies other than doxorubicin.

### 3.2. Vascular Toxicity Prevention

Few studies have investigated the effects of exercise on vascular function during breast cancer treatment. Six weeks of exercise training, initiated four weeks after doxorubicin treatment, was associated with improved endothelium-independent but not endothelium-dependent vasodilation, and with reduced mortality in rats with cardiac dysfunction [75]. Similarly, eight, but not four weeks of exercise training prior to exposure to 5-fluorouracil chemotherapy was associated with enhanced endothelium-dependent vasodilation in rats [52]. In humans, two small randomized trials of the effect of exercise training during doxorubicin-containing chemotherapy on endothelial function have had conflicting results [55, 70]. To advance understanding of exercise prevention of cardiovascular disease in breast cancer survivors, future exercise cardioprotection studies should include measurement of vascular function in addition to the cardiac measures.

### 3.3. Cardiovascular Risk Factors Modification

Traditional cardiovascular risk factors should be monitored and managed in breast cancer patients who receive cardiotoxic cancer therapies to prevent additional injury [76]. Exercise can favorably improve a number of cardiovascular risk factors including hypertension, raised cholesterol/lipids, overweight and obesity, raised blood glucose or diabetes, and cardiorespiratory fitness [77].

Hypertension is more than twice as prevalent among breast cancer survivors aged 55 and older as it is among the general population [78], and may be caused by chemotherapy agents used to treat breast cancer including cyclophosphamide, cisplatin and carboplatin [79]. Chemotherapy for breast cancer is also associated with elevations in triglyceride levels [80], while tamoxifen treatment may reduce levels of protective high density lipoprotein (HDL) [81]. Prior to treatment, breast cancer survivors may already have a suboptimal lipid profile including higher total cholesterol, triglyceride, and low density lipoprotein levels, and lower HDL levels than healthy controls [82–86]. A similar pattern occurs with overweight or obesity, where overweight, a risk factor for development of breast cancer [87], is often an issue prior to treatment, and chemotherapy treatment perpetuates the problem via its association with greater weight gains than other treatments in the year following diagnosis [88]. Therefore, it is not surprising that almost half of breast cancer survivors are overweight or obese [89]. Treatment also has lasting adverse effects on peak oxygen consumption (VO_2_), the gold standard measurement of cardiorespiratory fitness [90]. Chemotherapy causes a 6–10% reduction in peak VO_2_ [71, 91], and following breast cancer treatment completion, remains an average of 22% lower than that of healthy sedentary controls [92]. Furthermore, the level of cardiorespiratory fitness amongst breast cancer survivors appears to mediate incidence of cardiovascular disease and risk factors [93]. Lastly, breast cancer survivors are at an increased risk for diabetes from two up to 10 years following diagnosis [94], and its presence increases the risk of mortality in this population [95]. In early stage breast cancer survivors, high blood insulin levels, indicative of insulin resistance, are associated with obesity, poor lipid profiles [96], distant recurrence and death [97].

A number of exercise intervention studies in human breast cancer survivors have included cardiovascular risk factors as outcome measures. Exercise interventions in breast cancer survivors have consistently reported decreases in systolic blood pressure of 3–5 mmHg both during [98–100] and after [99, 101–105] treatment. Reported effects on blood lipids following an exercise intervention with or without dietary intervention include significant positive effects on triglycerides [102, 105], and HDL [105], or no effect [104, 106, 107]. Numerous exercise interventions have measured weight or body composition change with mixed results, showing either no effect or weight reduction [12]. Small feasibility studies have demonstrated that the combination of exercise with a diet intervention could be more effective in reducing weight in breast cancer survivors [106, 107]. Exercise training during chemotherapy or radiation treatment for breast cancer at minimum can prevent the peak VO_2_ decline occurring in usual care controls [71], or improve peak VO_2_ [70, 72, 91, 108, 109]. Exercise training following completion of breast cancer treatment improves peak VO_2_ [106, 110, 111]. Only one [105] of six randomized controlled trials to examine the effect of an exercise intervention on insulin and/or insulin resistance demonstrated statistically significant changes [104, 107, 112–114]. This same study also reported improvements in fasting blood glucose [105].

In summary, exercise interventions appear to have clinically meaningful effects on blood pressure and peak VO_2_, whereas the effects on blood lipids, weight, and insulin/glucose and potential development of diabetes are less clear. The strong established relationships between both blood pressure and peak VO_2_ and cardiovascular disease development and mortality in noncancer populations [6, 115–117] provide convincing support for the role of exercise in prevention of cardiovascular disease in human breast cancer survivors.

## 4. Translation of Preclinical Findings to Clinical Studies

Substantial preclinical evidence supports the role of exercise in prevention of cardiovascular disease toxicity, and there is some evidence for modification of cardiovascular risk factors in clinical trials. Further clinical research is warranted to determine whether exercise is a feasible and effective method for the reduction of cardiovascular morbidity and mortality in breast cancer survivors. Barriers to the translation of preclinical findings to human models include the need for more sensitive outcome measures and uncertainty regarding the optimal exercise dose.

Demonstration of the cardioprotective benefits of exercise in rodents has typically required euthanasia. One of the greatest barriers to this research in humans is identification of a noninvasive and sensitive outcome measure. Three-dimensional echocardiography-derived LVEF has emerged as a more reliable measure of LV function in patients receiving chemotherapy compared to traditional two-dimensional imaging [118], although this does not necessarily imply greater sensitivity to early changes in function. Echocardiography-derived LV global longitudinal strain and strain rate are able to detect changes in cardiac function during chemotherapy, radiation and trastuzumab treatment before changes in LVEF are detectable [119]. In noncancer populations, cardiac strain responds to exercise training [120]. Our research group is conducting an ongoing study to determine whether exercise training can prevent the doxorubicin-related decline in cardiac strain parameters in women with breast cancer. These parameters are widely available in conjunction with standard echocardiography [121]; with acceptable inter- and intra-observer variability (5% and 3.5%, resp.) [122]. Global longitudinal strain is predictive of all-cause mortality for a number of other cardiac conditions [123–126], and may be a stronger predictor of outcomes than LVEF [123, 126], but its relationship with clinical outcomes other than LVEF in breast cancer survivors is unknown.

Endothelial function is another attractive clinical outcome measure because dysfunction is an early process in the development of cardiovascular disease, and in noncancer populations, responds to pharmacological [42, 127] and exercise [50] interventions. Endothelial function can be easily measured in humans with a reactive hyperemia test, in which a cuff is inflated around the arm to occlude blood flow for 5 minutes. With release, the sudden increase in blood flow causes vasodilatation, which can be measured with ultrasound or peripheral arterial tonometry [127].

Cardiac biomarkers may play a role in predicting and identifying cardiotoxicity [128]. N-terminal prohormone brain natriuretic peptide (NT-proBNP) is frequently elevated during and after anthracycline treatment in adults [129–131]. There is mixed evidence regarding its ability to predict cardiac dysfunction following anthracycline treatment [130–132], as several studies where trastuzumab treatment followed anthracycline treatment, do not report a predictive ability of NT-proBNP [122, 133–135]. Due to inter-individual variations in kinetics, several measurements may be required to capture an elevation in cardiac troponins in patients receiving anthracyclines [122, 129, 130, 133, 134, 136–146], but the occurrence of an elevation in troponin I is predictive of chemotherapy and trastuzumab-related decreases in LVEF [139, 147], and cardiac events [140]. Exercise in heart failure patients does not change levels of NT-proBNP [148] or cardiac troponin I [149], but chronic heart failure has a different pathophysiology than the acute effects of cardiotoxic cancer therapies. Nonetheless, cardiac biomarkers may prove to be an effective outcome measure for exercise cardioprotection interventions due to their accessibility and reliability as a marker of cardiotoxicity.

Another important factor in the effective translation of preclinical findings to humans is the exercise intervention design. While preclinical and clinical experimental studies demonstrate that high intensity aerobic exercise results in greater cardiac benefits than moderate or low intensity [150, 151], the strenuous exercise prescription applied in most preclinical studies (five days a week, moderate to high intensity, 20–90 minutes) would likely not be tolerable for humans undergoing chemotherapy treatment [152]. One rodent study implemented a more clinically feasible and practical exercise prescription and doxorubicin treatment protocol involving 20 minutes of low intensity exercise, performed five days per week during chronic low dose doxorubicin treatment [23]. Although the lower doxorubicin dose failed to induce the MHC isoform shift and lipid peroxidation reported with higher doses, the lower exercise dose was protective against LV dysfunction and cardiomyocyte apoptosis [23]. In heart failure patients, moderate intensity exercise performed three days per week has been shown to improve systolic function [153]. Therefore, the required exercise dose for cardioprotection likely involves three to five days per week of moderate to high intensity aerobic exercise of at least 20 minutes in duration, but greater benefits will likely occur with higher doses. The optimal prescription requires a balance of patient tolerance with protective efficacy.

## 5. Conclusion

Breast cancer therapy has efficacious antitumor effects, but is associated with increased risk of cardiovascular disease. A considerable body of research, including preclinical studies and clinical trials, indicates that exercise may be an effective nonpharmacological method of attenuating the harmful effects of breast cancer therapies on the heart and vasculature, of modifying cardiovascular risk factors, and potentially reducing cardiovascular morbidity and mortality in this vulnerable population. The mechanisms for exercise prevention appear to be predominantly related to an increase in antioxidant capacity and associated reduction in oxidative stress. Clinical trials are needed to investigate the role of exercise in the prevention of direct cardiovascular toxicity of breast cancer treatment and the effect on cardiovascular events and mortality. The role of exercise in the prevention of cardiovascular disease in other cancer populations also warrants further research, as the detrimental combination of a high incidence of baseline risk factors combined with cancer treatment cardiovascular toxicity may be common to multiple cancer types. Echocardiographic quantification of LV global longitudinal strain and strain rate, endothelial function quantification, and measurement of circulating cardiac biomarkers are safe, noninvasive measures that may be sensitive and effective outcome measures for clinical studies of exercise prevention of breast cancer treatment-related cardiovascular toxicity. The exercise frequency, intensity, and duration demonstrating cardioprotection in most preclinical studies may need to be modified to accommodate human patient tolerability during ongoing cancer treatment.

## Figures and Tables

**Table 1:** Potential mechanisms for exercise prevention of doxorubicin-related cardiovascular toxicity.

Myocardial target	Role of target	Direction of exercise-induced change^*^	Direction of doxorubicin-induced change^*^	Evidence of exercise prevention of doxorubicin-induced change
Mechanisms with evidence for their role in exercise prevention				
Antioxidant to oxidative stress ratio	Prevention of oxidative damage	↑ [15]	↓ [13]	✓ [17–21, 52] × [23]
Expression of *α* : *β* myosin heavy chain isoform in rodents	Motor protein required for muscular contraction; in a healthy rodent heart there is a much higher concentration of the *α* isoform	↑ [154]	↓ [155]	✓ [28, 35–37] × [23]
Caspase 3 and 9 activity	Markers for apoptotic signaling	↓ [156]	↑ [14]	✓ [18, 23, 24, 26]
HSP 60 expression	Controls protein folding and unfolding in response to stress	↑ [18]	↑↑ [19]	✓ [17, 19]
Mitochondrial permeability transition pore opening	Regulation of calcium handling and apoptosis	↓ [157]	↑ [158]	✓ [26]
Ubiquitin-proteasome activation	Maintains protein function and quality control	↓ [159]	↑ [160]	✓ [24]
Endothelial progenitor cell level	Physiologic and pathologic vessel formation	↑ [54]	↓ [161]	✓ [55]
HSP72 expression	Controls protein folding and unfolding in response to stress	↑ [18, 162]	= [163]	✓ [23] × [24, 40]
SERCA2a expression	Calcium recycling from the cytosol into the sarcoplasmic reticulum	↑ [164]	↓ [165]	✓ [166] × [28, 29]

Mechanisms with evidence against their role in exercise prevention				
HSP 70 expression	Controls protein folding and unfolding in response to stress	↑ [167]	↓ [168]	× [19]
AMPK activation	Senses and regulates energy homeostasis	↑ [169]	↓ [170]	× [166]
Cardiac progenitor cell level/heart mass	Physiological turnover of cardiomyocytes	↑ [171]	↓ [172]	× [29]
Expression of PGC-1*α*	Transcription coactivator that regulates mitochondrial biogenesis and angiogenesis	= [32, 33]	↓ [173]	× [22, 34]

Potential mechanisms for exercise prevention lacking investigation				
Neuregulin-1/ErbB4 signalling	Cardiac cell survival growth factor	↑ [60]	↓ [174]	*∅*
Expression of GATA-4	Transcription factor involved in cardiac survival, hypertrophic growth of the heart	↑ [58]	↓ [175]	*∅*

↑: increase; ↓: decrease; =: no change; ✓: evidence available in favor of this mechanism; ×: evidence available against this mechanism; *∅*: no evidence available.

HSP: heat shock protein; SERCA: sarcoplasmic reticulum calcium pump; AMPK: AMP-activated protein kinase; PGC: peroxisome proliferator-activated receptor-γ coactivator.

^*^Note: Where possible reference cited provides evidence for the cardiomyocyte response, which may differ from other cell types.
